# Virtual reality-induced emotion recognition with deep learning-based multimodal physiological feature fusion

**DOI:** 10.3389/fpsyg.2026.1759471

**Published:** 2026-04-15

**Authors:** Xiaoli Fan, Chaoyi Zhao, Hua Guo, Feng Wu, Xin Zhang

**Affiliations:** 1 Air Force Medical Center, PLA, Beijing, China; 2 Key Laboratory of Human Factors and Ergonomics (HF&E), State Administration for Market Regulation, Beijing, China

**Keywords:** emotion recognition, long short-term memory, multimodal fusion, physiological signal, principal component analysis, virtual reality

## Abstract

**Objective:**

Recognizing emotions objectively and accurately remains challenging because of the limited ecological validity, informational incompleteness, and constrained model performance of conventional approaches. This study addresses these limitations holistically by investigating a novel framework that integrates ecologically valid virtual reality (VR) for emotion elicitation with deep learning-based multimodal physiological signal fusion.

**Methods:**

An immersive VR environment was developed to effectively elicit three target emotional states: positive, neutral, and negative. Synchronized physiological signals—electroencephalography (EEG), electrocardiography (ECG), and galvanic skin response (GSR)—were recorded from 20 healthy participants alongside subjective self-assessment data. After preprocessing and feature extraction, a nested cross-validation procedure was employed to prevent data leakage: within each of the five folds, feature selection (one-way repeated-measures ANOVA, *α* = 0.05) was performed solely on the training data. A hybrid network architecture combining principal component analysis (PCA) with long short-term memory (LSTM) was employed for dimensionality reduction and modeling. The PCA retained components explaining 90% cumulative variance, while the LSTM layer contained 96 hidden units, followed by three fully connected layers with integrated dropout regularization. Model performance was evaluated using this rigorous cross-validation framework and compared against baseline models including support vector machine (SVM), random forest (RF), k-nearest neighbors (k-NN), and extreme gradient boosting (XGBoost).

**Results:**

Subjective evaluation results confirmed the effectiveness of VR-induced emotion elicitation. At the group level, one-way repeated-measures analysis of variance revealed significant main effects of emotional states (*p* < 0.05) on multiple physiological features: EEG frontal alpha asymmetry indices (AI_F4/F3, AI_F8/F7), ECG indices (SDNN, RMSSD, LF/HF ratio, sample entropy), and GSR measures (SCL, NS.SCRs). Employing a nested cross-validation framework to prevent data leakage, the PCA-LSTM model achieved a mean accuracy of 87.18% ± 2.28% under five-fold cross-validation, significantly outperforming SVM (75.83% ± 4.25%), RF (78.89% ± 6.85%), k-NN (72.78% ± 5.21%), and XGBoost (81.67% ± 5.83%).

**Conclusion:**

This study validates that integrating an ecologically valid VR emotion elicitation paradigm with a multimodal PCA-LSTM fusion model effectively enhances the objectivity and accuracy of emotion recognition. The proposed framework provides an effective solution to overcome the bottlenecks of ecological validity and quantification precision in traditional methods, demonstrating preliminary application potential in intelligent human–computer interaction and mental-health monitoring domains.

## 1 Introduction

Human–computer interaction is undergoing a paradigm shift from functional implementation to intelligent collaboration (Deng et al., 2022). With the proliferation of technologies such as virtual reality (VR) and mobile computing, traditional command-centric interaction models have become increasingly inadequate for complex scenarios, making intelligence and naturalism the core development direction for next-generation human–machine systems (Xu et al., 2023). Against this backdrop, enabling machines to accurately perceive, understand, and respond to human emotional states has become a key scientific challenge for achieving intelligent human–machine collaboration. The core technology for achieving this breakthrough is affective computing (Picard, 1997): interpreting human emotional states via computational models allows machines to detect users’ emotional fluctuations, cognitive load, and even latent intentions, thereby facilitating a transition from passive response to proactive adaptation. Picard (2020) noted that “emotion is not the antithesis of reason but a crucial component of intelligent decision-making.” Affective computing not only fills the emotional gap in human–computer interaction but also provides a theoretical and technical foundation for building genuinely human-centric intelligent systems (Zhao et al., 2023). As the core component of affective computing, emotion recognition directly determines the performance of emotionally intelligent systems. Emotion recognition based on physiological signals demonstrates unique advantages in high-risk-industry human-factor safety and mental-health monitoring because it is objective and difficult to fake. For instance, in aviation and automotive driving, multimodal emotion recognition systems have become a key technology for enhancing human-factor safety: real-time monitoring of operators’ states can effectively warn of negative states such as emotional stress and distraction, thereby reducing accident risks significantly (Zhou D. et al., 2023; Zhou Y. et al., 2023). In mental-health monitoring, emotion recognition provides new avenues for the early screening and objective assessment of conditions such as depression and post-traumatic stress disorder: it enables non-invasive, continuous tracking of emotional states via wearable devices, thereby overcoming the limitations of traditional subjective scales (Lian et al., 2021).

Current research in emotion recognition revolves primarily around two technical approaches: external behavior-based (facial expression, speech, posture) and physiological signal-based (electroencephalography [EEG], electrocardiography [ECG], galvanic skin response [GSR], etc.). Behavior-based recognition is susceptible to interference from individual habits, cultural background, social masking, and environmental factors, often leading to insufficient accuracy and limited model generalizability (Li et al., 2022). In contrast, physiological signals regulated by the autonomic nervous system are difficult to disguise consciously, thereby providing a more objective and authentic reflection of an individual’s internal affective state (Candia-Rivera et al., 2022). This advantage makes physiological signal-based emotion recognition indispensable for high-reliability application scenarios. For example, Lu et al. (2022) and Zhang et al. (2021) successfully achieved high-accuracy recognition of driver fatigue and stress by collecting heart-rate variability (HRV) and GSR signals from wearable devices. Furthermore, Zhou D. et al. (2023) and Zhou Y. et al. (2023) used high-density EEG signals and graph neural networks to explore functional connectivity patterns in the brain during emotion elicitation, offering new tools for the precise assessment of emotional disorders. Recent research further indicates that multimodal physiological-signal fusion can significantly enhance the accuracy and robustness of emotion recognition (Ahmed and Khan, 2022; Shin et al., 2022).

However, physiological signal-based emotion recognition still faces three interconnected core challenges in practical applications. First, significant individual differences in physiological responses limit the generalizability of single-modality recognition models, while the effectiveness of multimodal fusion technology is heavily reliant on deep learning models that can mine complex nonlinear relationships. Traditional machine learning methods such as support vector machine (SVM) and random forest (RF) have shown clear limitations in this regard (Saganowski et al., 2022; Liu et al., 2022; Lu et al. (2022); Zhang et al., 2021). Second, most existing studies used simplified laboratory paradigms such as images or videos to induce emotions, resulting in limited ecological validity and substantial discrepancies from emotional experiences in real-world situations (Koelstra et al., 2012; Soleymani et al., 2012). Third, emotions are inherently dynamic, and experimentally induced emotional states are often transient. While temporal dynamics significantly impact recognition accuracy (Abadi et al., 2015), this characteristic has not been sufficiently emphasized or effectively modeled in existing research.

To address these challenges systematically, a comprehensive solution is proposed herein. The approach is first to use VR technology to construct a high-ecological-validity emotion elicitation paradigm, compensating for the ecological limitations of traditional methods and leveraging VR’s immersive characteristics to induce more authentic and intense emotional responses (Zhang M., 2023; Zhang S. et al., 2023; Zhang Z. et al., 2023; Pavic et al., 2023). Building on this, the research synchronously acquires multimodal physiological signals including EEG, ECG, and GSR based on the central-autonomic nervous system mechanism of emotion generation, overcoming model generalization challenges caused by individual differences through multi-source information complementarity (Gupta et al., 2023). Finally, to address the dynamic temporal characteristics of emotion, we design a PCA-LSTM hybrid model: utilizing principal component analysis (PCA) for dimensionality reduction of high-dimensional features to enhance model efficiency, while leveraging the exceptional temporal modeling capability of long short-term memory (LSTM) networks to capture the dynamic evolution patterns of emotions (Borghesi et al., 2023), ultimately achieving high-precision emotion state recognition. This study addresses systematically the current key bottlenecks in emotion recognition through the technical pathway of VR elicitation–multimodal acquisition–deep learning fusion, providing a new solution for objective, accurate, and reliable emotion recognition, and offering technical support for the applied development in fields such as intelligent human–computer interaction and mental-health monitoring.

## 2 Methods

### 2.1 Experimental design

Sixty-six candidate VR video resources were collected via academic databases such as Google Scholar and IEEE Xplore, as well as content platforms including YouTube 360° and SteamVR, using keywords such as “exciting,” “relaxing,” and “fearful.” The videos were screened and edited according to the following criteria: (i) the plot should be easily understandable without additional explanation; (ii) the targeted emotion (positive, negative, or neutral) elicited should be clear and singular; (iii) the duration should be no more than 2 min. The video clips were edited using VirtualDub (ver. 1.4.7) and processed uniformly into MPEG2 files with a resolution of 1920 × 1,080 pixels using Ulead VideoStudio 10.0.

**Figure 1:**
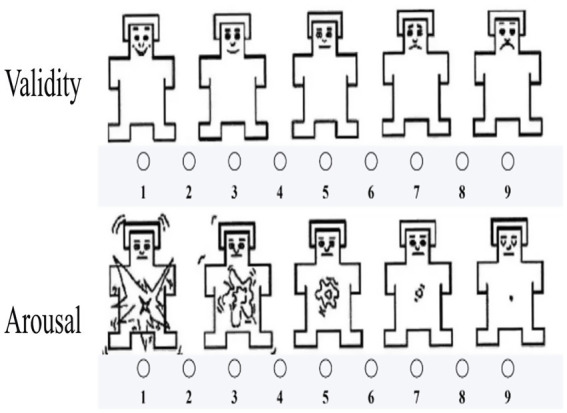
Nine-point Self-Assessment Manikin (SAM) scale.

Subsequently, 10 volunteers were invited for a preliminary experiment to rate the emotional valence and arousal elicited by the videos using the nine-point Self-Assessment Manikin (SAM) scale, as shown in Figure 1. Valence ranged from 1 (very unpleasant) to 9 (very pleasant), and arousal ranged from 1 (very calm) to 9 (very excited). In total, 30 valid video clips were ultimately obtained and categorized into three types: positive videos (valence > 7, arousal > 6), negative videos (valence < 3, arousal > 6), and neutral videos (valence = 4–6, arousal < 4).

In the formal experiment, the six most-effective video clips from each category (18 in total) were selected as stimulus materials and presented randomly via a Unity program. To ensure the transparency of the stimulus materials, Table 1 provides detailed characteristics of these 18 videos, including video ID, emotion category, theme type, core content description, duration, and the mean valence and arousal ratings (with standard deviations) obtained from the pre-experiment. The detailed content descriptions and pre-experiment ratings in Table 1 collectively ensure the representativeness of the selected stimuli. The video files are available from the corresponding author upon reasonable request for academic purposes.

**Table 1:** Characteristics of the 18 selected VR video clips.

ID	Emotion	Theme type	Core content description	Valence (Mean ± SD)	Arousal (Mean ± SD)
V01	Positive	Amusement park	Roller coaster first-person view, rapid motion, bright colors, upbeat background music	7.8 ± 0.6	7.2 ± 0.8
V02	Positive	Natural landscape	Tropical beach walk, sound of waves, sunny, relaxing atmosphere	8.1 ± 0.5	6.5 ± 0.7
V03	Positive	Animal fun	Pet dog playing, amusing actions, lighthearted and humorous	7.5 ± 0.7	6.8 ± 0.9
V04	Positive	Sports highlights	Soccer match highlight compilation, exciting commentary	7.9 ± 0.6	7.5 ± 0.7
V05	Positive	Music performance	Pop concert live, enthusiastic audience	7.6 ± 0.8	7.0 ± 0.8
V06	Positive	Travel scenery	Drone footage of mountains and lakes, magnificent scenery	7.7 ± 0.6	6.2 ± 0.8
V07	Neutral	Daily scene	Office routine, people typing and filing documents	5.1 ± 0.4	3.2 ± 0.5
V08	Neutral	Life scene	Kitchen cooking, ordinary family activities	5.3 ± 0.5	3.5 ± 0.6
V09	Neutral	City street	Ordinary street with pedestrians walking, no special events	5.0 ± 0.4	3.1 ± 0.5
V10	Neutral	Indoor environment	Quiet library, people reading	4.9 ± 0.5	2.8 ± 0.5
V11	Neutral	Natural scene	Ordinary park scenery, no strong emotional elements	5.4 ± 0.5	3.3 ± 0.6
V12	Neutral	Traffic scene	Ordinary road traffic, no accidents or abnormalities	5.2 ± 0.4	3.4 ± 0.5
V13	Negative	Horror scene	Dark forest, sudden frightening appearance	2.5 ± 0.5	7.8 ± 0.7
V14	Negative	Disaster scene	Earthquake ruins, tense rescue operations	2.3 ± 0.4	7.5 ± 0.8
V15	Negative	Medical emergency	Hospital emergency room, tense scene	2.8 ± 0.6	7.0 ± 0.8
V16	Negative	Horror film	Classic horror movie clip, suspenseful	2.1 ± 0.4	8.0 ± 0.7
V17	Negative	War scene	War documentary clip, explosions	2.4 ± 0.5	7.6 ± 0.8
V18	Negative	Psychological thriller	Suspenseful chase scene, tense atmosphere	2.6 ± 0.5	7.4 ± 0.7

**Figure 2:**
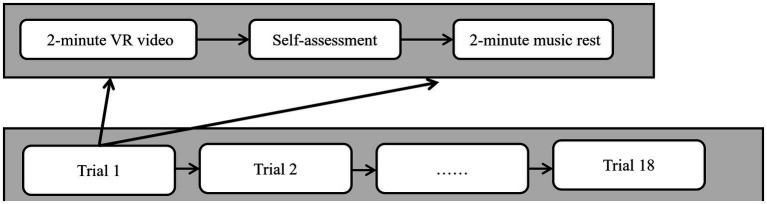
Experimental procedure for emotion induction.

The experimental procedure is illustrated in Figure 2. Each trial consisted of a 2-min VR video viewing, an immediate SAM scale rating, and a 2-min music rest phase to ensure emotional recovery to baseline. This design ensured the validity of emotion elicitation, the randomness of the experimental sequence, and effective control of interference between trials.

In the formal experiment, a total of 360 trials were collected (20 participants × 18 trials each). The trials were perfectly balanced across the three target emotional states, with 120 trials per class (positive, neutral, negative). This trial-wise sample constituted the fundamental dataset for the subsequent machine learning analysis. In the formal experiment, a total of 360 trials were collected (20 participants × 18 trials each). The trials were perfectly balanced across the three target emotional states, with 120 trials per class (positive, neutral, negative). This trial-wise sample constituted the fundamental dataset for the subsequent machine learning analysis.

### 2.2 Participants and data acquisition

Twenty healthy participants (10 males and 10 females; mean age = 23.8 ± 2.4 years) were recruited through university-wide advertisements. The inclusion criteria were (a) normal or corrected-to-normal vision; (b) no history of neurological, psychiatric, or cardiovascular disorders, (c) a resting heart rate of between 60 and 100 beats per minute, (d) no prior experience of VR-induced motion sickness, and (e) normal emotional state as evaluated by the Profile of Mood States-Short Form administered prior to the experiment. Among the participants, 7 had prior VR experience while 13 were VR-naïve. The mean resting heart rate was 72.5 ± 5.6 bpm. They were instructed to maintain regular sleep–wake cycles and abstain from alcohol, caffeine, and other stimulants for 24 h before the experimental session. On the day of testing, they were also asked to wash their hair thoroughly to ensure high-quality EEG signal acquisition. All participants provided written informed consent, were informed explicitly of their right to withdraw from the study unconditionally at any point, and received monetary compensation upon completion of the experiment.

**Figure 3:**
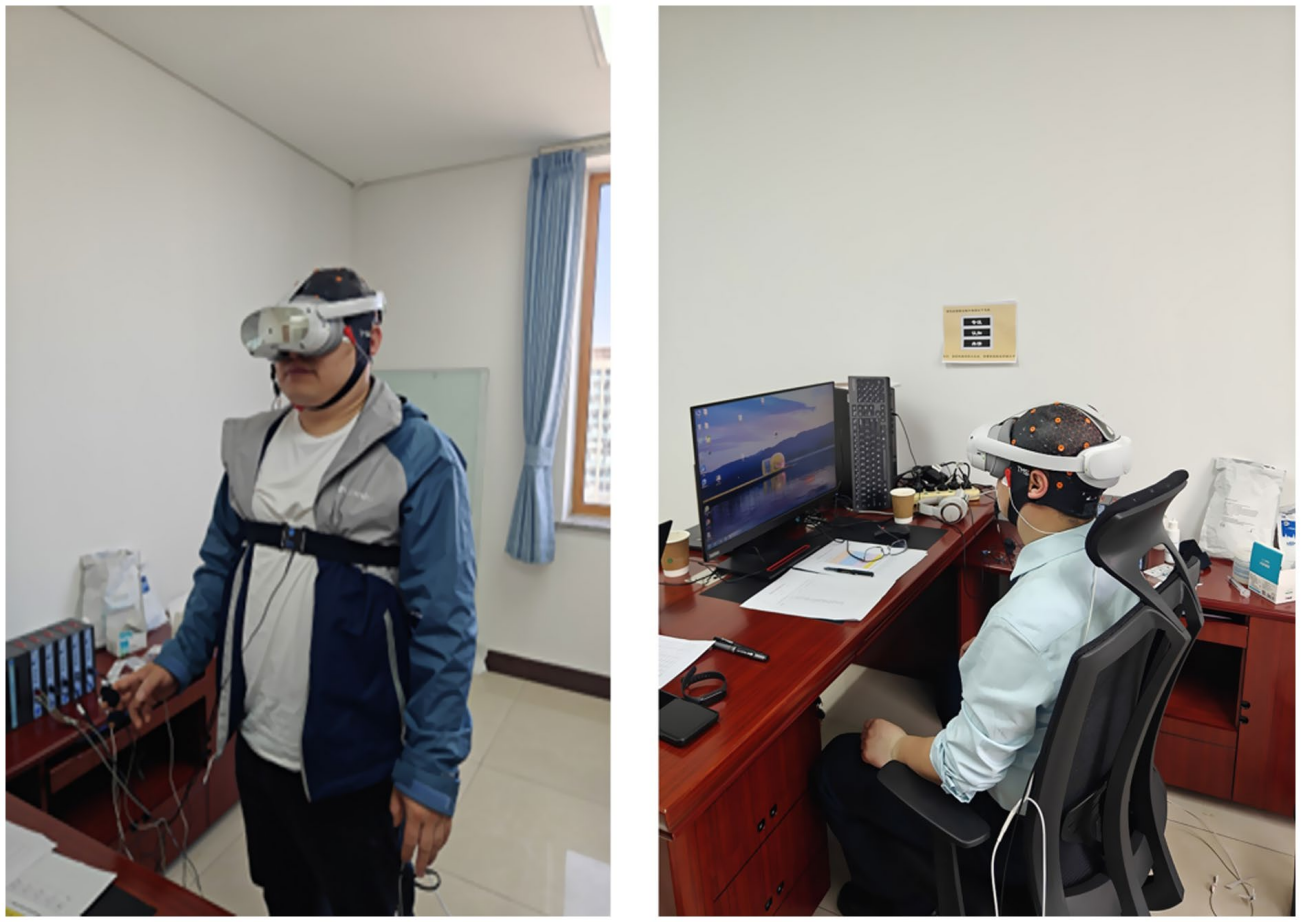
Experimental session in progress.

The experiment was conducted in a dedicated soundproofed and electromagnetically shielded laboratory with strictly controlled environmental parameters: temperature = 22 °C ± 1 °C, humidity = 50% ± 5%, background noise < 35 dB, and background illumination ≈ 2 cd/m^2^. Stimuli were presented using a PICO 4 Ultra VR headset. Physiological signals were recorded using a SAGA 32 EEG system for six prefrontal channels (FP1, FP2, F3, F4, F7, F8) and an MP160 multi-channel physiological recorder for synchronized acquisition of ECG and GSR signals. The specific parameter settings were as follows: EEG signals were sampled at 512 Hz with AC coupling, a gain of 1,000, and bandpass filtering of 0.05–100 Hz; both ECG and GSR signals were sampled at 500 Hz, with ECG recorded using a three-lead configuration with disposable Ag-AgCl electrodes and bandpass filtering of 0.5–35 Hz. Stimulus presentation and data acquisition were synchronized via TTL trigger signals sent from the stimulus program to the recording systems at the onset of each trial. Before data collection, participants completed a 10-min adaptation period to familiarize themselves with the VR environment, and signal quality was checked prior to each trial. The experimental setup is shown in Figure 3.

### 2.3 Signal preprocessing

The acquired raw physiological signals underwent the following modal-specific preprocessing pipelines to remove artifacts and prepare for feature extraction.

EEG: The continuous EEG data were first re-referenced to the average reference of the six prefrontal channels. This prefrontal focus is grounded in the well-established prefrontal asymmetry theory of emotion, which associates asymmetric frontal activity with emotional valence. In addition to the hardware bandpass filtering (0.05–100 Hz), a 50 Hz notch filter was applied to suppress power-line interference. Independent Component Analysis (ICA) was then performed using the EEGLAB toolbox to identify and remove components corresponding to ocular artifacts (blinks and saccades), cardiac activity, and persistent muscle noise. Potential artifacts from the VR headset were mitigated by ensuring minimal head movement and inspecting for device-specific noise in the power spectrum. Bad channels, identified by visual inspection and abnormal impedance (>10 kΩ), were excluded from analysis. The data were subsequently epoched into 2-min segments corresponding to each VR stimulus. Epochs or channels containing residual high-amplitude artifacts were rejected based on a ± 100 μV threshold and visual inspection. This comprehensive pipeline ensured the analysis was conducted on clean, artifact-minimized EEG data.

ECG/HRV and GSR: For ECG, the signal was processed using the Pan-Tompkins algorithm for R-peak detection, following the hardware bandpass filtering (0.5–35 Hz). HRV features were selected based on their established sensitivity to autonomic nervous system dynamics during emotional processing. Time-domain indices (SDNN, RMSSD) reflect overall HRV and parasympathetic activity; frequency-domain indices (LF, HF, LF/HF ratio) capture sympathetic-parasympathetic balance; and sample entropy quantifies the complexity of cardiac regulation. The derived RR-interval series was visually inspected and automatically corrected: intervals that differed from the preceding interval by more than 20% were labeled as ectopic beats and corrected via linear interpolation. This resulted in a clean normal-to-normal (NN) interval series for each 2-min trial. All HRV metrics (SDNN, RMSSD, LF, HF, LF/HF ratio, and sample entropy) were computed from the entire 2-min NN-interval series. For GSR, the signal was low-pass filtered at 0.3 Hz and then Z-score normalized within each participant’s data to account for individual differences in baseline skin conductance. GSR features (SCL and NS.SCRs) were selected as they are well-validated indicators of sympathetic arousal. Skin Conductance Responses (SCRs) were detected automatically using the Ledalab toolbox with a minimum amplitude threshold of 0.01 μS.

### 2.4 Feature extraction and selection

Features were then extracted from the preprocessed signals described in Section 2.3. Specifically, EEG signals were analyzed using wavelet packet analysis, performing five-level decomposition on three pairs of homologous prefrontal electrodes (FP1/FP2, F3/F4, F7/F8). Based on the reconstructed power in the alpha frequency band (8–13 Hz), three asymmetry indices were calculated using the formula AI = ln(P_α_Right) − ln(P_α_Left). ECG signals were bandpass filtered (0.5–40 Hz) and processed using the Pan–Tompkins algorithm for R-wave detection and RR interval series construction. Six HRV features were extracted, comprising time-domain (SDNN, RMSSD), frequency-domain (LF, HF, LF/HF ratio), and nonlinear (sample entropy) indices. GSR signals were low-pass filtered (0.3 Hz) and Z-score normalized, followed by extraction of two key time-domain features: skin conductance level and non-specific skin conductance response frequency.

One-way repeated-measures analysis of variance (ANOVA) was conducted as an exploratory physiological analysis to assess the general sensitivity of the extracted features to emotional states using the entire dataset. Greenhouse–Geisser correction was applied when necessary, and post-hoc tests were conducted using Tukey’s HSD (honestly significant difference) method. This group-level analysis precedes the fold-specific feature selection process described in the next section for model development.

### 2.5 Dimensionality reduction and classification based on feature fusion

To ensure the rigor and unbiasedness of the model’s generalization performance evaluation, this study employed an analysis procedure nested within the stratified five-fold cross-validation framework. Specifically, within each fold of the cross-validation, the feature screening step was performed independently: ANOVA was executed solely on the training set of that fold to screen for physiological features exhibiting significant differences across emotional states. Subsequently, only the subset of features selected from that fold’s training set was used for subsequent modeling.

To achieve complementary information integration across modalities, the EEG, ECG, and GSR features screened within each fold were concatenated at the feature level to construct a fold-specific unified multimodal feature set. PCA is an unsupervised linear dimensionality reduction technique. Given the fused feature matrix X ∈ ℝ^(*n* × p)^, PCA computes the covariance matrix C = (1/(*n*-1))X^T^ X and performs eigen decomposition C = VΛV^T. The reduced feature set is obtained as Z = XW_k, where W_k contains the top k eigenvectors. The cumulative variance explained by the first k components is:


(∑{i=1}^kλ_i)/(∑{i=1}^pλ_i)

Where λ_i are the eigenvalues. In this study, k was selected such that the cumulative variance reached 90%. To handle the high dimensionality and temporal characteristics of these fused features, a hybrid network architecture combining PCA and LSTM was developed. This architecture employs PCA for dimensionality reduction of the fused features (cumulative variance threshold: 90%) to eliminate redundancy, while utilizing LSTM to capture their temporal dependencies. LSTM model temporal dependencies through gating mechanisms. At each time step t, the hidden state h_t is updated as:


f_t=σ(W_f⋅[h_(t−1),x_t]+b_f)

i_t=σ(W_i⋅[h_(t−1),x_t]+b_i)

c~_t=tanh(W_c⋅[h_(t−1),x_t]+b_c)

c_t=f_t⊙c_(t−1)+i_t⊙c~_t

o_t=σ(W_o⋅[h_(t−1),x_t]+b_o)

h_t=o_t⊙tanh(c_t)

**Figure 4:**
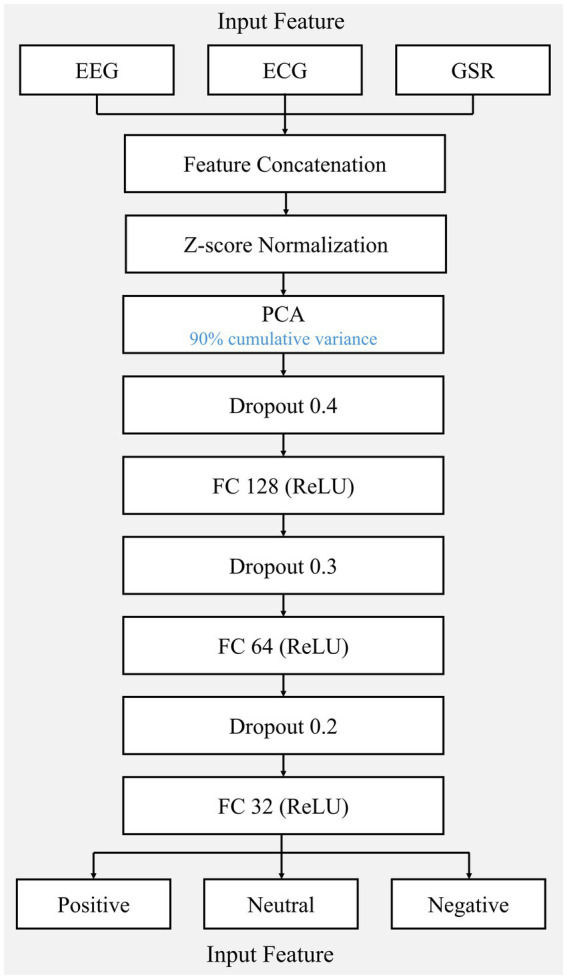
Architecture of the proposed PCA-LSTM model.

Where σ is the sigmoid function, ⊙ denotes element-wise multiplication, and W and b are learnable weights and biases. The sequential integration of PCA and LSTM is theoretically motivated by their complementary roles: PCA reduces dimensionality and eliminates feature redundancy, while LSTM captures the temporal dynamics of emotional states. The network consists of a single LSTM layer (96 hidden units) followed by three fully connected layers (128–64–32 neurons), with progressively decreasing dropout rates (0.4–0.3–0.2) applied to hidden layers to prevent overfitting. The model was trained using the Adam optimizer (learning rate: 0.001) with dynamic learning-rate adjustment and early stopping mechanisms. Through the aforementioned stratified five-fold cross-validation, with feature screening strictly embedded within each fold, the dataset was partitioned into training, validation, and test sets (7:1.5:1.5 ratio) to ultimately accomplish the three-class (positive, neutral and negative). All experiments were conducted on a standard deep learning workstation using Python with TensorFlow, along with EEGLAB, MNE-Python, and Ledalab toolboxes for signal processing. Figure 4 illustrates the complete architecture of the proposed PCA-LSTM model, depicting the flow from input feature sequences through PCA dimensionality reduction, LSTM temporal modeling, fully connected layers with dropout regularization, to final emotion classification.

## 3 Results

### 3.1 Analysis of emotion elicitation validity

To evaluate the effectiveness of the VR emotion elicitation paradigm, a one-way repeated-measures ANOVA was conducted on the subjective report data from all 360 trials (20 participants × 3 emotions × 6 videos). The results revealed significant main effects of emotion type in both valence and arousal dimensions [valence: *F*(2, 38) = 68.44, *p* < 0.05, η^2^ = 0.78; arousal: F(2, 38) = 35.67, p < 0.05, η^2^ = 0.65]. *Post-hoc* analyses using Tukey’s HSD method demonstrated a clear graded relationship in valence ratings: positive > neutral > negative emotions (all ps < 0.05). For arousal, both positive and negative emotions showed significantly higher ratings than the neutral condition (all ps < 0.05), while no significant difference was found between positive and negative emotions (*p* = 0.215). These findings confirm that the VR-based elicitation paradigm effectively induced the theoretically expected emotional states, thereby providing a reliable foundation for subsequent physiological signal analysis.

### 3.2 Analysis of multimodal physiological features

#### 3.2.1 EEG features

To identify effective EEG representation indicators for emotion recognition, statistical analysis was conducted on three prefrontal asymmetry indices (AI_Fp2/Fp1, AI_F4/F3, AI_F8/F7). One-way repeated-measures ANOVA revealed significant main effects of different emotional conditions on the dorsolateral prefrontal indicators AI_F4/F3 (*F*(2, 38) = 9.24, *p* < 0.05, η^2^ = 0.871) and AI_F8/F7 (F(2, 38) = 7.86, *p* < 0.05, η^2^ = 0.871), while the main effect for the frontopolar indicator AI_Fp2/Fp1 did not reach statistical significance (F(2, 38) = 1.16, *p* = 0.32, η^2^ = 0.65).

**Figure 5:**
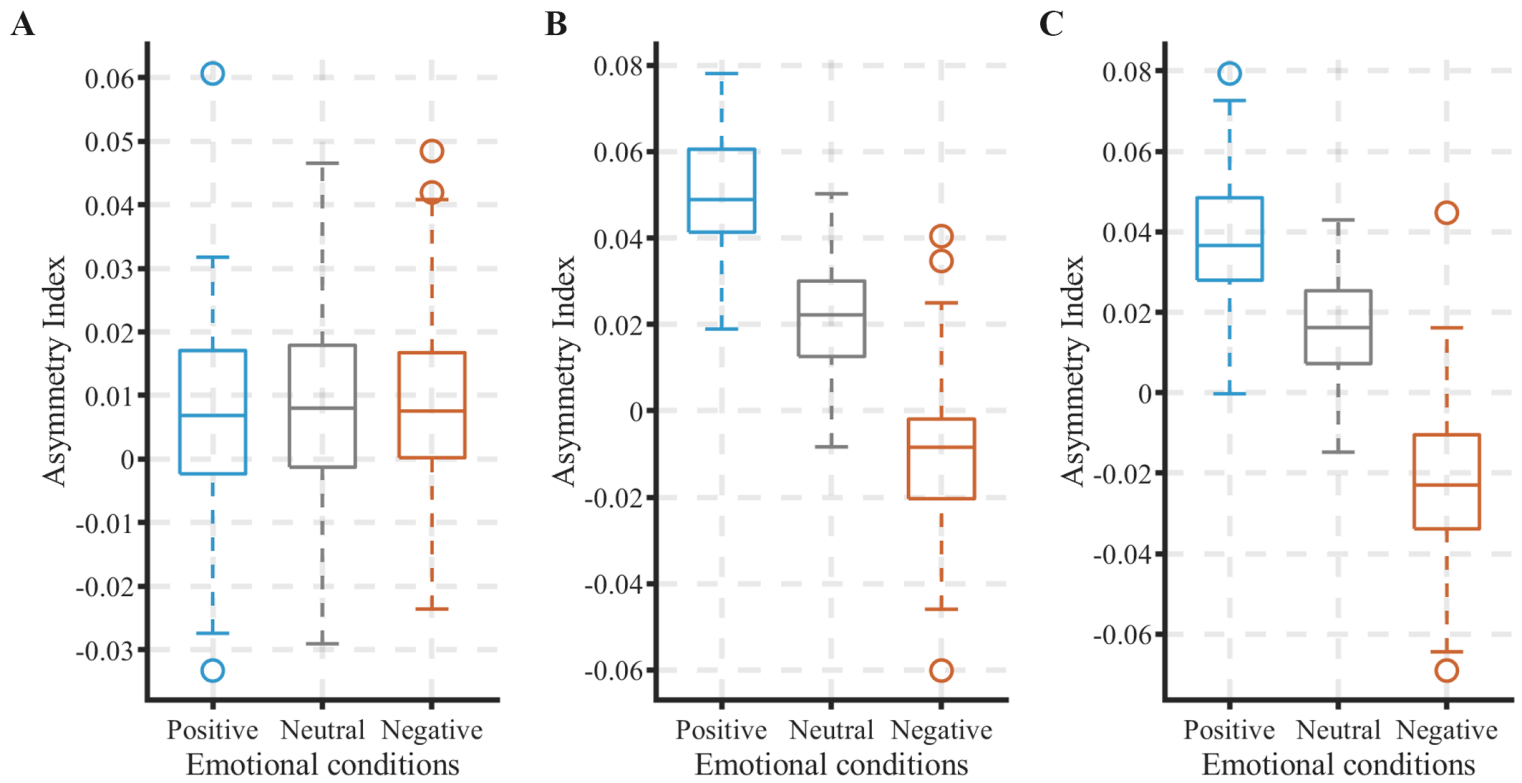
Comparison of prefrontal alpha-band asymmetry indices across three emotional conditions: **(A)** AI-Fp2/Fp1 **(B)** AI-F4/F3 **(C)** AI-F8/F7.

As shown in Figure 5, boxplot analysis visually demonstrates the emotion discrimination capability of the three indicators. For both AI_F4/F3 and AI_F8/F7, the box distributions show a clear emotional gradient: the AI values are highest for positive emotions, intermediate for neutral emotions, and lowest for negative emotions. This distribution pattern is fully consistent with post-hoc test results: for these two effective indicators, values during positive emotions are significantly higher than during both neutral (ps < 0.05) and negative emotions (ps < 0.05), while values during negative emotions are significantly lower than during neutral emotions (ps < 0.05). Therefore, AI_F4/F3 and AI_F8/F7 demonstrated a significant capacity to differentiate among the induced emotional states.

#### 3.2.2 ECG features

This study evaluated the emotion representation capability of six electrocardiographic features through a multi-indicator analysis of HRV. One-way repeated-measures ANOVA revealed significant main effects of emotional states on the time-domain indices SDNN [*F*(2, 38) = 12.47, *p* < 0.05, η^2^ = 0.84] and RMSSD [F(2, 38) = 8.92, *p* < 0.05, η^2^ = 0.78], the frequency-domain index of LF/HF ratio [F(2, 38) = 15.63, *p* < 0.05, η^2^ = 0.89], and the nonlinear index of sample entropy [F(2, 38) = 10.28, *p* < 0.05, η^2^ = 0.82]. In contrast, between-group differences for LF power (*p* = 0.087) and HF power (*p* = 0.124) did not reach statistical significance.

**Figure 6:**
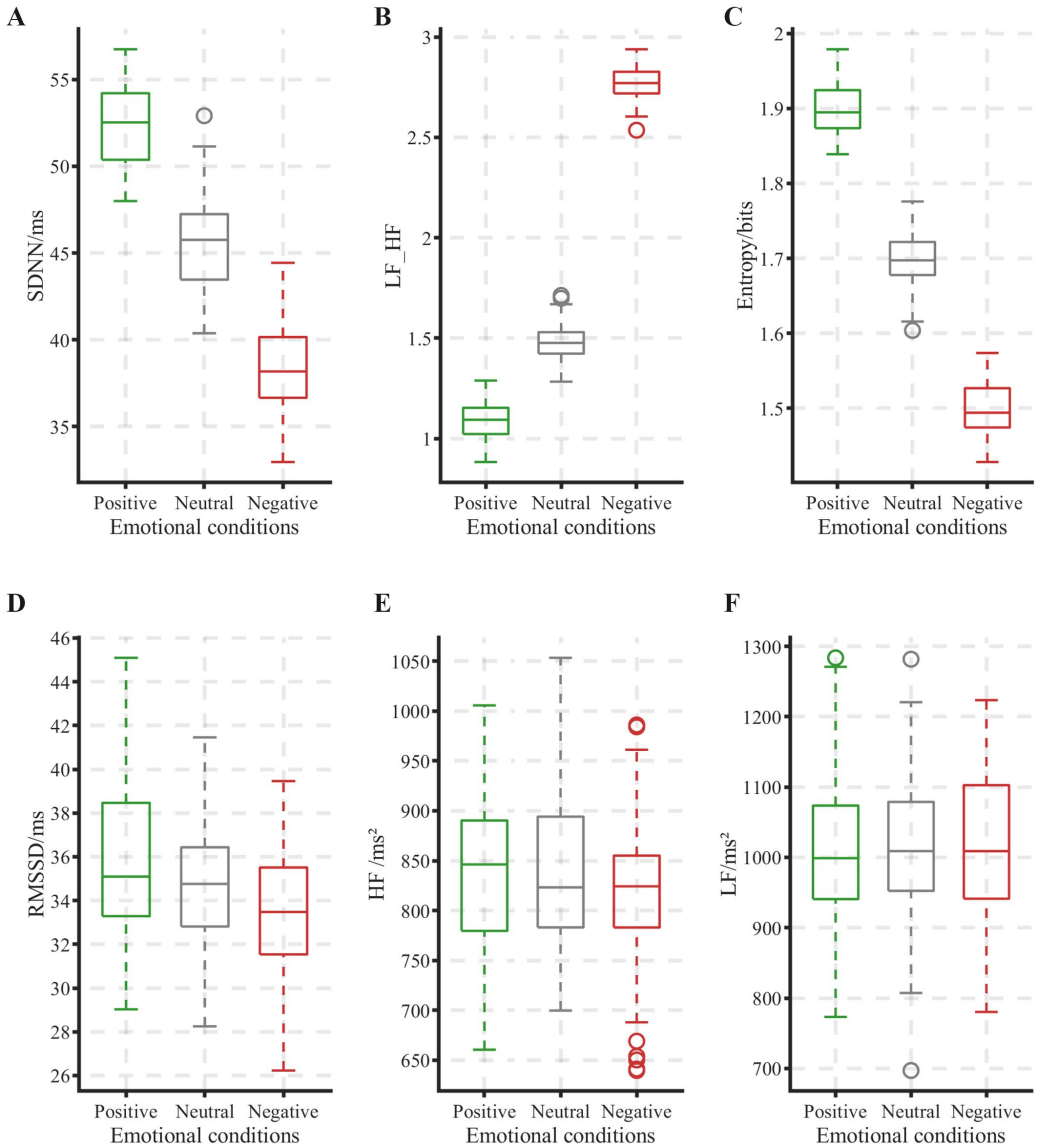
Comparison of heart-rate variability indices across three emotional conditions. **(A)** SDNN; **(B)** LF-HF; **(C)** entropy; **(D)** RMSSD; **(E)** HF; **(F)** LF.

As shown in Figure 6, boxplot analysis further revealed the emotion discrimination patterns of effective indicators. For time-domain measures, both positive and neutral emotions showed significantly higher SDNN and RMSSD values than for negative emotions, with minimal boxplot overlap. Post-hoc tests confirmed that positive and neutral emotions were significantly higher than negative emotions on both indices (all ps < 0.05), while no significant difference was observed between positive and neutral emotions. This pattern indicates that SDNN and RMSSD effectively characterize reduced autonomic nervous activity associated with negative emotions. In the frequency domain, the LF/HF ratio demonstrated a distinct pattern: values during negative emotions were significantly higher than during both positive and neutral conditions. Nonlinear analysis revealed significantly decreased sample entropy during negative emotions (*p* < 0.05), reflecting diminished physiological regulatory flexibility in negative emotional states. At the group level, ECG-derived indices—including time-domain (SDNN, RMSSD), frequency-domain (LF/HF ratio), and nonlinear (sample entropy) measures—were significantly sensitive to the induced emotional states.

#### 3.2.3 GSR features

**Figure 7:**
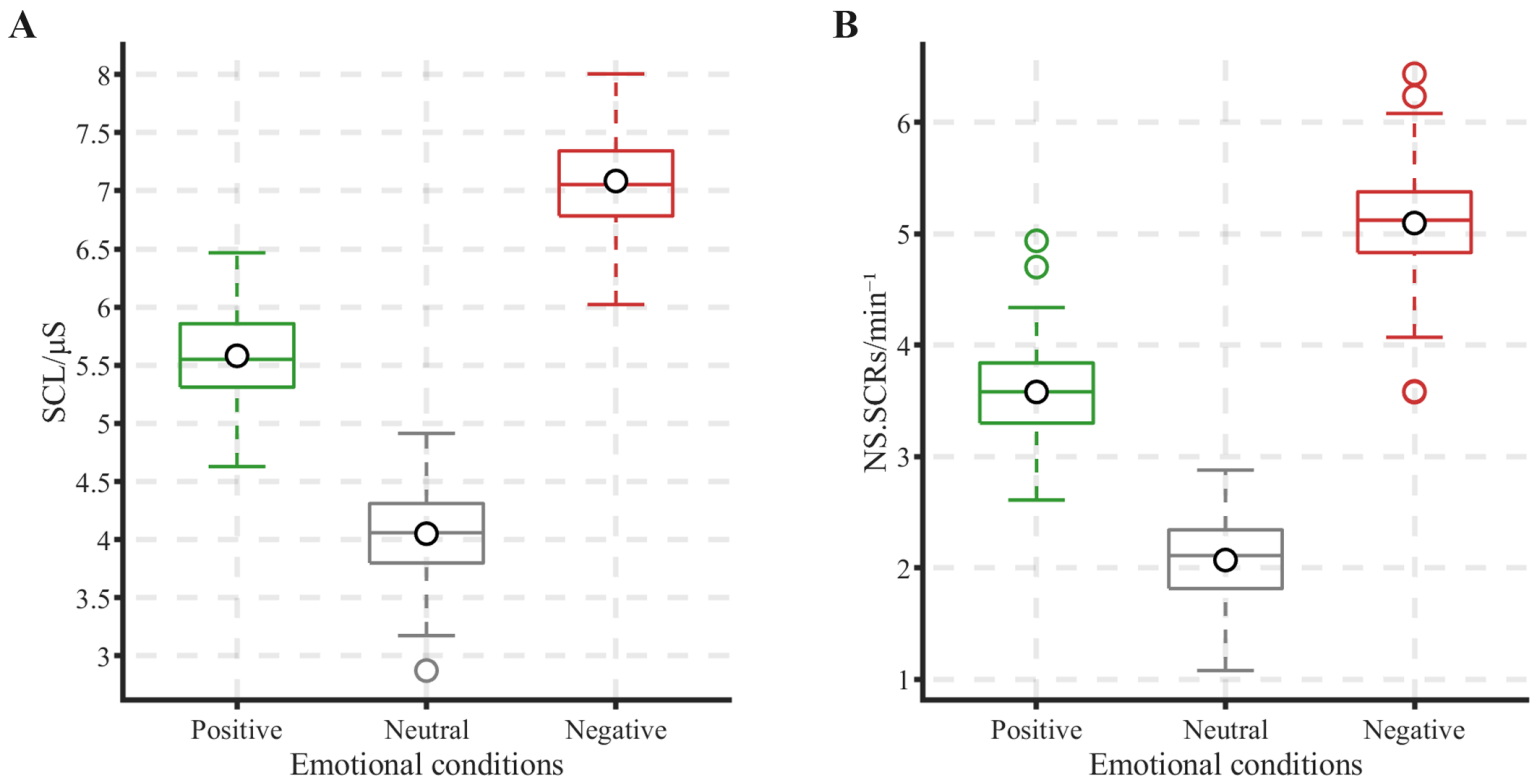
Comparison of galvanic skin response indices across three emotional conditions. **(A)** SCL **(B)** NS. SCR.

A one-way repeated-measures ANOVA was conducted on the GSR indicators SCL and NS.SCRs. The results revealed significant main effects of emotional conditions on both SCL (*F*(2, 38) = 600.08, *p* < 0.05, η^2^ = 0.97) and NS.SCRs (F(2, 38) = 396.48, *p* < 0.05, η^2^ = 0.95). As shown in Figure 7, boxplot distributions of both indicators demonstrated similar emotional response patterns. For both SCL and NS.SCRs, values during negative emotional conditions were significantly higher than during both positive and neutral conditions. Post-hoc tests further confirmed that values during negative emotions were significantly higher than during positive emotions (*p* < 0.05) and neutral conditions (*p* < 0.05), while a significant difference also existed between positive and neutral emotions (*p* < 0.05). These results indicate that both SCL and NS.SCRs differed significantly across emotional states, with the highest values observed during negative emotional conditions.

### 3.3 Multimodal feature fusion and classification

Building on the screened multimodal features, a feature-level fusion strategy was further employed to construct a PCA-LSTM fusion model for effective emotion state recognition and classification.

#### 3.3.1 Model performance evaluation metrics

To evaluate model performance, we employed standard metrics based on true positives (TP), true negatives (TN), false positives (FP), and false negatives (FN):


Accuracy=(TP+TN)/(TP+TN+FP+FN)

Precision=TP/(TP+FP)

Recall=TP/(TP+FN)

F1−score=2×(Precision×Recall)/(Precision+Recall)

MacroF1=(1/3)×(F1_pos+F1_neu+F1_neg)

All metrics were calculated for each cross-validation fold and reported as mean ± standard deviation.

#### 3.3.2 Feature dimensionality reduction effectiveness and overall model performance

**Figure 8:**
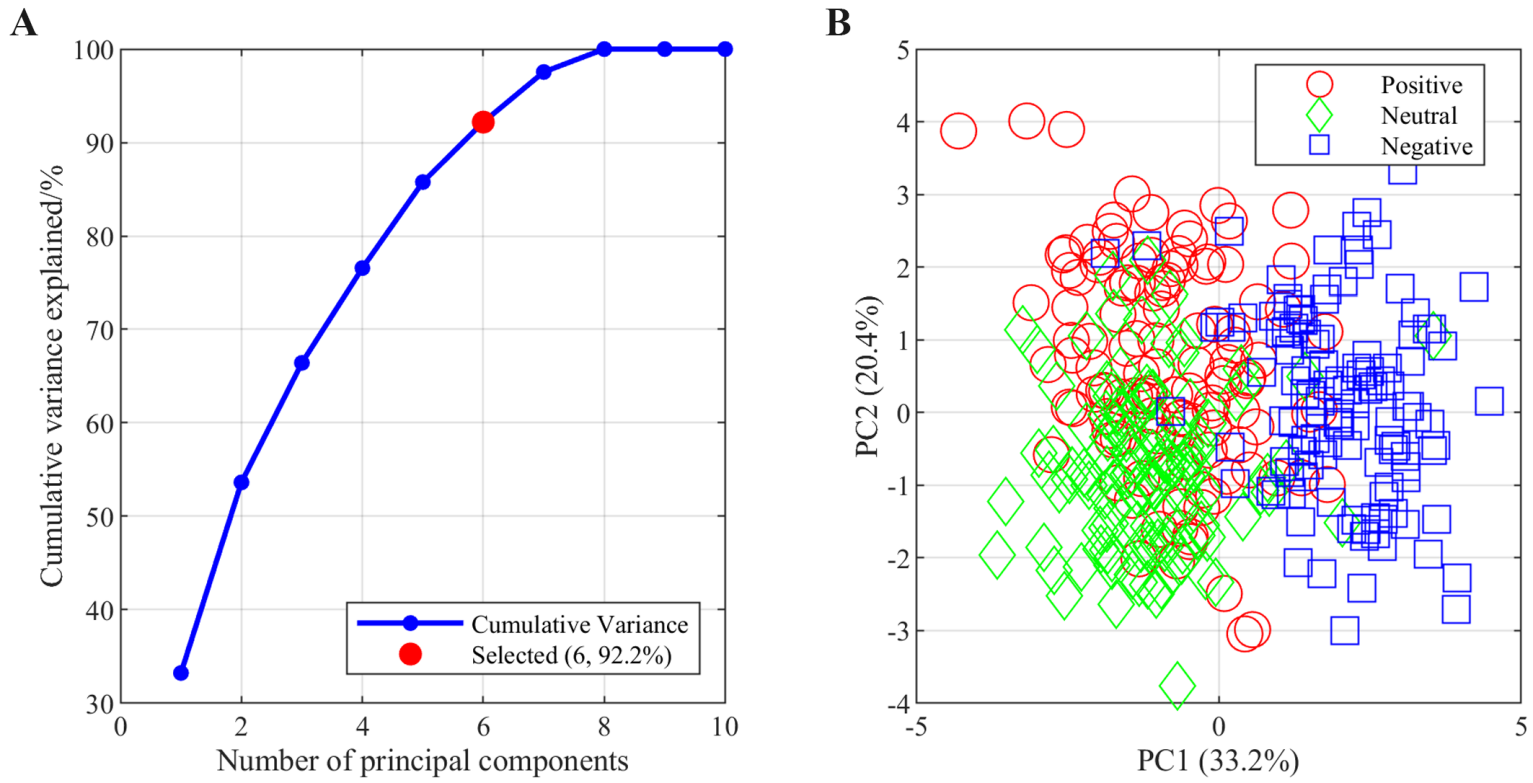
Dimensionality reduction analysis of fused multimodal features: **(A)** Cross-validation accuracy across folds and **(B)** Data projection on first two PCs.

Dimensionality reduction was performed on the fused multimodal features using PCA, which significantly enhanced model training efficiency while preserving most essential information. As shown in Figure 8A, the first six principal components retained 92.2% of the cumulative variance, effectively reducing feature dimensionality. Figure 8B displays the distribution of samples in the feature space constituted by the first two principal components, where different emotion categories demonstrate certain clustering tendencies and separability, indicating that the dimensionality-reduced fused features retain critical information for distinguishing emotional states.

**Figure 9:**
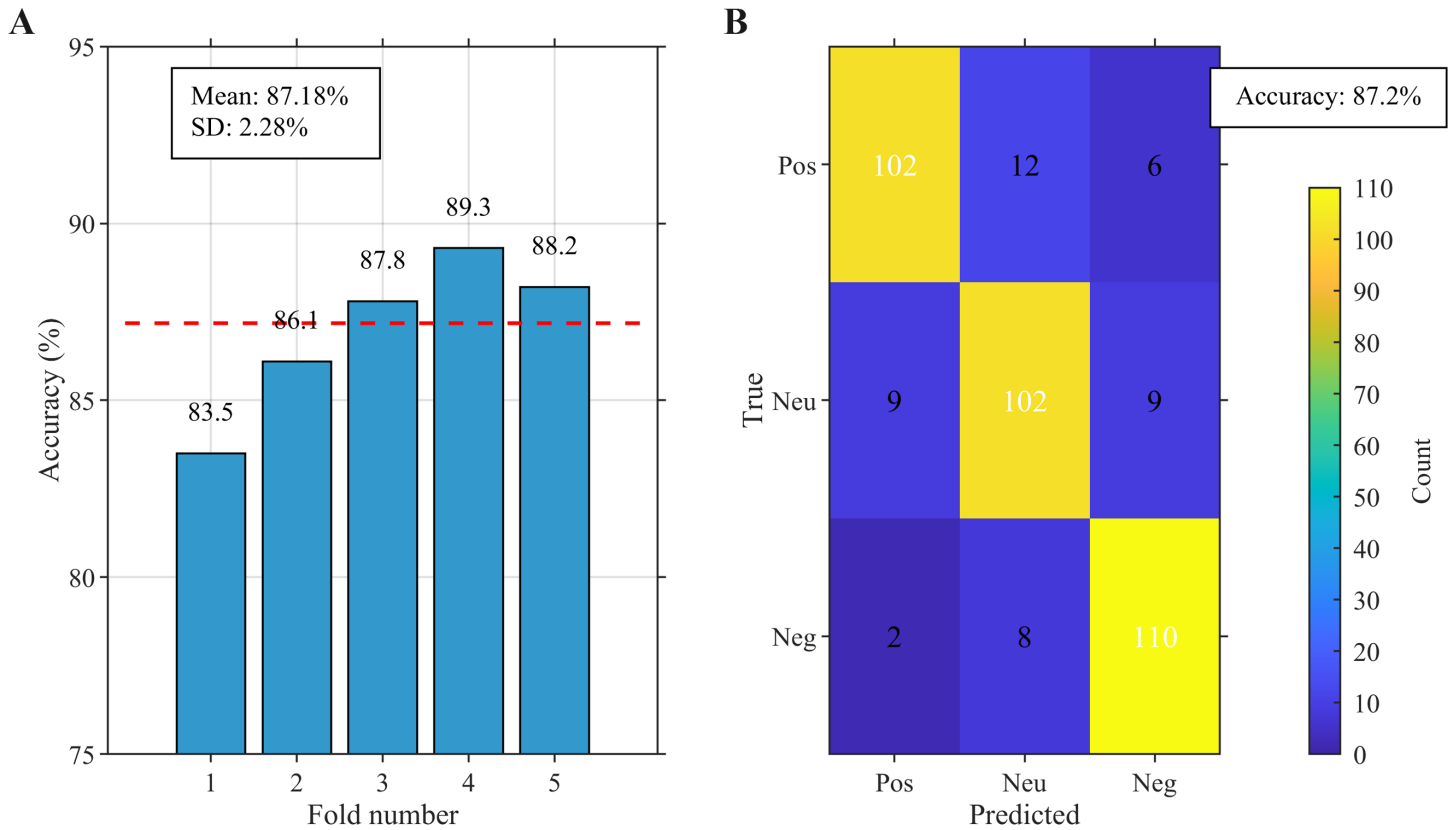
Model performance evaluation. **(A)** PCA variance explanation **(B)** Confusion matrix for emotion classification.

The PCA-LSTM model, evaluated under the nested cross-validation framework, demonstrated excellent performance in emotion recognition tasks. Using this rigorous evaluation, the model achieved a mean classification accuracy of 87.18% with a standard deviation of 2.28% across folds, indicating good stability. As shown in Figure 9A, the highest validation accuracy reached 89.3% (fold 4), while the lowest was 83.5% (fold 1), demonstrating strong robustness to different data partitions. The confusion matrix in Figure 9B further reveals balanced recognition performance across the three emotion categories. Quantitative metrics for each emotion class are detailed in Table 2. Negative emotions achieved the highest recall (91.7%), while positive and neutral emotions attained recalls of 85.0 and 85.0%, respectively. This performance hierarchy aligns with the physiological response patterns observed in Section 3.2, where negative emotions elicited the most pronounced autonomic signals.

**Table 2:** Per-class performance metrics of the PCA-LSTM model.

Emotion class	Precision	Recall	F1-Score	Support (Trials)
Positive	90.3%	85.0%	87.5%	120
Neutral	83.6%	85.0%	84.3%	120
Negative	88.0%	91.7%	89.8%	120
Macro Average	87.3%	87.2%	87.2%	360
Weighted Average	87.3%	87.2%	87.2%	360

#### 3.3.3 Training process analysis and comparative validation

**Figure 10:**
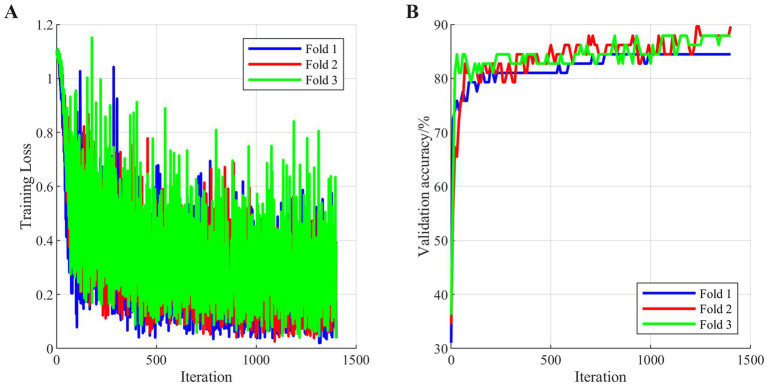
Model training consistency. **(A)** Training loss across folds **(B)** Validation accuracy across folds.

The model training process exhibited excellent stability and convergence. As shown in Figure 10, the training curves across different folds showed high consistency. The training losses converged rapidly without significant oscillation, while the validation accuracy curves demonstrated that all folds reached stable levels during mid-training with similar final convergence values. This consistency in the training process reflects both the rationality of the model architecture and hyperparameter settings, and it indicates that the PCA-reduced fused features provide a stable and high-quality learning foundation for the model.

**Figure 11:**
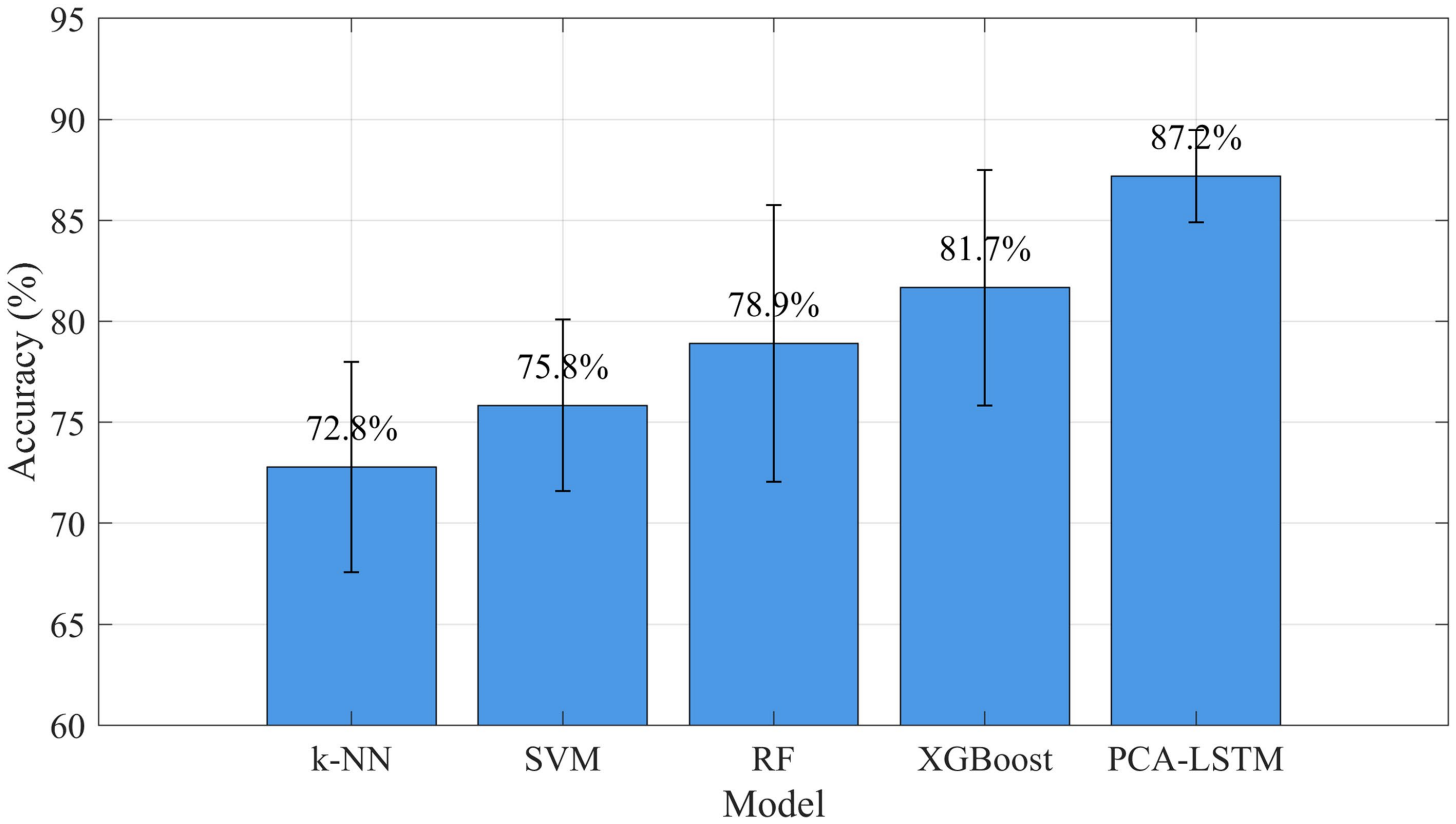
Performance comparison of different models.

Comparative results with classical machine learning methods are shown in Figure 11. We compared the proposed PCA-LSTM model with four baseline models: SVM, RF, k-NN, and XGBoost. All models were evaluated using the same nested cross-validation framework. The PCA-LSTM fusion model achieved optimal classification performance (87.18% ± 2.28%), significantly outperforming SVM (75.83% ± 4.25%), RF (78.89% ± 6.85%), k-NN (72.78% ± 5.21%), and XGBoost (81.67% ± 5.83%). Paired-sample t-tests with Bonferroni correction confirmed these differences were statistically significant (Table 3). Meanwhile, PCA-LSTM demonstrated smaller performance variation in cross-validation (standard deviation: 2.28%), indicating the model’s excellent robustness.

**Table 3:** Results of paired-sample *t*-tests comparing model performance.

Comparison	Difference (%)	95% CI	*t*	*p*	p_adj	*d*
PCA-LSTM vs. RF	8.28	[3.24, 13.32]	4.48	0.011	0.022	1.96
PCA-LSTM vs. SVM	11.34	[7.47, 15.21]	7.94	0.001	0.002	2.73
PCA-LSTM vs. XGBoost	5.51	[1.82, 9.20]	3.84	0.018	0.054	1.42
PCA-LSTM vs. k-NN	14.40	[9.85, 18.95]	8.52	<0.001	<0.001	3.12

*Adjusted *p*-values account for multiple comparisons (4 tests) using Bonferroni correction.

## 4 Discussion

This study systematically constructed an emotion recognition framework based on multimodal physiological signals. Through VR emotion elicitation, multimodal feature fusion, and deep learning modeling, accurate identification of three basic emotional states was achieved. The research results validate the effectiveness of the technical approach, reflected primarily in the following three aspects.

First, the VR-based emotion elicitation paradigm demonstrated good ecological validity in this study. Subjective report data revealed significant gradient differences in the valence dimension across the three emotional states (positive > neutral > negative). In the arousal dimension, high-arousal emotions (positive and negative) were significantly higher than the neutral emotion. These findings are consistent with previous research on emotion elicitation (Chirico et al., 2016; Zhang M., 2023; Zhang S. et al., 2023; Zhang Z. et al., 2023), confirming that VR technology can effectively induce target emotions and providing a reliable foundation for physiological signal analysis. Second, the multimodal physiological feature analysis revealed complementary mechanisms of different signals in emotional representation. For EEG features, the dorsolateral prefrontal asymmetry indices (AI_F4/F3, AI_F8/F7) showed sensitivity to emotional valence, validating the crucial role of the prefrontal cortex in emotional processing (Davidson, 2004; Li et al., 2023). At the autonomic nervous system level, the decrease in HRV indices (SDNN, RMSSD), increase in LF/HF ratio, and reduction in sample entropy during negative emotions collectively reflected a typical pattern of sympathetic activation and decreased physiological regulatory flexibility (Hu and Gao, 2022). The simultaneous rise in skin conductance level further corroborated this multi-system synergistic response mechanism (Kreibig, 2010; Chang et al., 2025). Building on these findings, the PCA-LSTM fusion model proposed in this study demonstrated significant advantages in emotion recognition. By effectively integrating the selected EEG, ECG, and GSR features through feature-level fusion, combined with PCA dimensionality reduction (retaining 92.2% variance information) and LSTM temporal modeling (Hochreiter and Schmidhuber, 1997; Liu et al., 2023), the model achieved 87.18% recognition accuracy, significantly outperforming traditional machine learning methods (Kim et al., 2023; Du et al., 2024). The cross-validation standard deviation of only 2.28% reflects good generalization capability. These results confirm the effectiveness of multimodal feature fusion in enhancing emotion recognition performance (Zhang M., 2023; Zhang S. et al., 2023; Zhang Z. et al., 2023).

It is important to acknowledge certain limitations of this study. Although the total number of independent observation samples (360 trials) provided a solid basis for the cross-validated analysis, the demographic profile of our participant sample (*N* = 20, homogeneous and healthy) may limit the immediate generalizability of the findings to broader populations. Future work should therefore focus on external validation with larger, more diverse cohorts to further establish the robustness of the proposed framework. Additionally, exploring end-to-end deep learning models for raw signal processing and translating the framework into real-time, adaptive VR applications represent promising directions for both research and practical implementation.

## 5 Conclusion

This study innovatively integrated VR-based emotion elicitation, multimodal physiological signal analysis, and deep learning modeling to establish a comprehensive affective computing framework. Through feature-level fusion strategy and the PCA-LSTM hybrid architecture, we validated the effectiveness of multimodal feature fusion in emotion recognition, providing a promising framework and novel insights for future research and potential applications in affective computing.

This study has several limitations. First, the homogeneous participant sample (*N* = 20) limits the demographic generalizability of our findings. It is critical to distinguish this from the internal validity of the model evaluation, which was rigorously conducted on 360 independent trials. Therefore, external validation with larger, more diverse cohorts is the essential next step. Second, while the controlled laboratory environment ensured data quality, it differs significantly from real-world complex settings. Additionally, the focus on three basic emotion states requires further exploration of complex emotions and continuous emotional intensity variations. Finally, the black-box nature of deep learning models compromises interpretability, thereby affecting result credibility and clinical acceptability.

Based on the current findings and limitations, future work should focus on the following.

*Data enhancement.* Expanding both the scale and diversity of datasets to include participants from varied age groups, cultural backgrounds, and clinical populations is crucial, as highlighted by challenges in building representative datasets (Xie et al., 2024). Concurrently, developing robust methods for naturalistic physiological signal acquisition will be vital for improving real-world applicability (Wang et al., 2024).

*Technical advancement.* Exploring advanced model architectures such as attention mechanisms to enhance recognition of key physiological features (Liu et al., 2024) or graph neural networks to model complex nonlinear relationships among multimodal features (Zhu et al., 2024) could significantly improve performance. A parallel focus should be on developing specialized interpretation tools to increase model transparency and foster trust (Yang et al., 2022).

## Data Availability

The raw data supporting the conclusions of this article are not publicly available due to privacy/ethical restrictions. Requests to access the datasets should be directed to the corresponding author.
